# Impact of Kidney Donor Profile Index Scores on Post-Transplant Clinical Outcomes Between Elderly and Young Recipients, A Multicenter Cohort Study

**DOI:** 10.1038/s41598-020-64055-8

**Published:** 2020-04-24

**Authors:** Woo Yeong Park, Jeong Ho Kim, Eun Jung Ko, Ji-Won Min, Tae Hyun Ban, Hye-Eun Yoon, Young Soo Kim, Kyubok Jin, Chul Woo Yang, Seungyeup Han, Byung Ha Chung

**Affiliations:** 1 Transplant research center, Department of Internal Medicine, Seoul St. Mary’s Hospital, College of Medicine, The Catholic University of Korea, Seoul, Republic of Korea; 2 Division of Nephrology, Department of Internal Medicine, Seoul St. Mary’s Hospital, College of Medicine, The Catholic University of Korea, Seoul, Republic of Korea; 3 Division of Nephrology, Department of Internal Medicine, Daejeon St. Mary’s hospital, College of Medicine, The Catholic University of Korea, Daejeon, Republic of Korea; 4 Division of Nephrology, Department of Internal Medicine, Bucheon St. Mary’s hospital, College of Medicine, The Catholic University of Korea, Bucheon, Republic of Korea; 5 Division of Nephrology, Department of Internal Medicine, Eunpyeong St. Mary’s hospital, College of Medicine, The Catholic University of Korea, Seoul, Republic of Korea; 6 Division of Nephrology, Department of Internal Medicine, Incheon St. Mary’s hospital, College of Medicine, The Catholic University of Korea, Incheon, Republic of Korea; 7 Division of Nephrology, Department of Internal Medicine, Uijeongbu St. Mary’s hospital, College of Medicine, The Catholic University of Korea, Uijeongbu, Republic of Korea; 8 Department of Internal Medicine, Keimyung University School of Medicine, Daegu, Republic of Korea; 9 Keimyung University Kidney Institute, Daegu, Republic of Korea

**Keywords:** End-stage renal disease, End-stage renal disease

## Abstract

We investigated if clinical outcomes after kidney transplantation (KT) from deceased donors (DDs) with high Kidney Donor Profile Index (KDPI) can be different according to the age of KT recipients (KTRs). Six-hundred fifty-seven KTRs from 526 DDs were included from four transplant centers. We divided KTRs into elderly-KTR and young-KTR groups based on age 60 and each group was subdivided into high- or low-KDPI subgroup based on KDPI score of 65%. We compared short-term and long-term clinical outcomes among those four subgroups (low KDPI-young KTR, low KDPI-elderly-KTR, high KDPI-young-KTR, high KDPI-elderly-KTR). In short-term outcomes including acute rejection, BK virus and CMV infection, there was no significant difference among the four subgroups. In the long-term outcomes, the development of cardiovascular disease was higher in the high KDPI-elderly-KTR group than the other groups. In comparison of allograft survival rate, the high KDPI-young KTR subgroup showed highest risk for allograft failure and there was significant interaction between high-KDPI donors and young-KTR on allograft survival rate (P = 0.002). However, there was no significant difference in comparison of the patient survival rate. In conclusion, clinical impact of high-KDPI in DDs on post-transplant allograft survival may be less significant in elderly-KTR than in young-KTR.

## Introduction

The number of elderly patients with end-stage renal disease (ESRD) has increased globally parallel to the increases in mean life expectancy over the past several decades^1,2^. According to the Korean ESRD Registry data, only 8% of the total number of ESRD patients were older than age 60 in 2004, which increased rapidly to 43.9% in 2016. Accordingly, the proportion of elderly patients undergoing kidney transplantation (KT) showed an almost six-fold increase, from 2% in 2000 to 11.6% in 2016^3^. In terms of clinical outcomes, KT offers a better quality of life, cardiovascular stability, and improved survival compared to peritoneal dialysis (PD) or hemodialysis (HD) in elderly ESRD patients; hence, several studies have suggested that patients should not be excluded from KT merely because of elderly age^4,5^.

Meanwhile, aging is associated with significant biological and metabolic changes that can affect their response to the allograft after KT. For example, elderly recipients have lower metabolism and less muscle mass and they also are less physically active, which may result in less nephron mass that can balance the work required of kidneys in elderly patients^6^. Additionally, the entire immune response is usually decreased in elderly recipients and they display altered pharmacokinetics, which can diminish the immune response to an allograft^7,8^. In this regard, kidneys from marginal donors that have reduced baseline kidney capacity may be a good match for the required work of kidneys in elderly recipients after KT. Thus, it is possible that the long-term clinical outcomes of KT from marginal donors can be a better match for elderly recipients than for young recipients who may require relatively more kidney mass^9,10^. However, this hypothesis has not been fully investigated.

Therefore, the purpose of this study was to investigate if KT from marginal deceased donors (DDs) with high Kidney Donor Profile Index (KDPI) scores showed different clinical outcomes in elderly recipients compared to younger recipients. For this, we compared the clinical outcomes of KTs according to the KDPI scores in DDs and the age of kidney transplant recipients (KTRs). We also determined if high KDPI scores in DDs showed an interaction with recipient age using established multicenter cohort data^11–14^.

## Results

### Comparison of clinical and laboratory parameters according to the KDPI score in deceased donors and the age of kidney transplant recipients

The proportion of DDs with high KDPI scores was significantly higher in the elderly KTR group than in the young KTR group (65.6% vs. 44.1%, *P* < 0.001). However, there was no significant difference in the distribution of donor gender; mean body mass index (BMI); and DDs with hypertension (HTN), diabetes mellitus (DM), death due to cerebrovascular accident (CVA), or baseline estimated glomerular filtration rate (eGFR) of DDs between the elderly KTR and the young KTR groups. There was also no significant difference in the distribution of recipient gender; mean BMI; and KTRs with HTN, DM, re-transplantation, duration of dialysis before KT, cold ischemic time, or high panel reactive antibody (PRA) (>50%) between the elderly KTR and the young KTR groups (Table S1).

In comparison of the baseline characteristics among the four subgroups (the low KDPI-young KTR, low KDPI-elderly KTR, high KDPI-young KTR and high KDPI-elderly KTR subgroups), the proportion of male gender, BMI, HTN, DM, dialysis vintage, the number of previous KT, cold ischemic time, the rate of induction and main immunosuppressant, and the proportion of high PRA ( > 50%) did not differ (Table 1).

**Table 1:** Comparison of clinical and laboratory parameters according to KDPI score and the age of KTRs.

Variables	Low KDPI- young KT	Low KDPI- elderly KT	High KDPI-young KT	High KDPI-elderly KT	p for Trend
**Donors**	n = 239	n = 30	n = 196	n = 61	
Age at KT (years)	35.6 ± 12.3	34.2 ± 12.5	53.8 ± 8.9	59.1 ± 7.5	<0.001
Gender (Male: Female)	173: 66	24: 6	132: 64	39: 22	0.292
Body mass index (kg/m^2^)	23.0 ± 4.1	23.3 ± 3.7	23.2 ± 3.3	22.9 ± 2.7	0.457
Hypertension, n (%)	9 (3.8)	2 (6.7)	76 (38.8)	20 (32.8)	<0.001
Diabetes mellitus, n (%)	6 (2.5)	0	34 (17.3)	10 (16.4)	<0.001
Cause of donor death - CVA, n (%)	151 (63.2)	16 (53.3)	145 (74.0)	51 (83.6)	0.001
Baseline GFR (ml/min/1.73m^2^) (CKD-EPI)	116.7 ± 35.7	117.9 ± 44.9	117.7 ± 28.7	118.9 ± 30.0	0.726
Acute kidney injury, n (%)	95 (39.7)	18 (60.0)	141 (71.9)	39 (63.9)	<0.001
**Recipients**	n = 285	n = 34	n = 262	n = 76	
Transplant year, n (%)					0.012
1996 ~ 2005	8 (2.8)	0	0	0	
2006 ~ 2010	51 (17.9)	3 (8.8)	37 (14.1)	4 (5.3)	
2011 ~ 2017	226 (79.3)	31 (91.2)	225 (85.9)	72 (94.7)	
Age at KT (yr)	45.7 ± 8.4	63.7 ± 2.7	47.8 ± 8.5	63.6 ± 3.2	<0.001
Gender (Male: Female)	168: 117	20: 14	155: 107	46: 30	0.997
Body mass index (kg/m^2^)	22.9 ± 4.1	23.9 ± 3.1	23.4 ± 3.6	23.1 ± 3.1	0.312
Hypertension, n (%)	232 (81.4)	31 (91.2)	224 (85.5)	66 (86.8)	0.348
Diabetes mellitus, n (%)	46 (16.1)	9 (26.5)	64 (24.4)	18 (23.7)	0.063
Dialysis duration, years	9.0 ± 9.0	7.0 ± 3.7	8.2 ± 13.0	6.9 ± 4.3	0.355
Previous KT, n (%)	41 (14.4)	4 (11.8)	22 (8.4)	3 (3.9)	0.023
Cause of ESRD, n (%)					<0.001
Glomerulonephritis	149 (52.3)	7 (20.6)	104 (39.7)	38 (50.0)	
Diabetes mellitus	35 (12.3)	9 (26.5)	51 (19.5)	19 (25.0)	
Hypertension	37 (13.0)	8 (23.5)	60 (22.9)	10 (13.2)	
Others	64 (22.5)	10 (29.4)	47 (17.9)	9 (11.8)	
Cold ischemic time (min)	255.3 ± 127.4	245 .2 ± 149.9	246.0 ± 119.3	254.9 ± 117.1	0.836
HLA mismatch number	3.5 ± 1.5	3.4 ± 1.6	3.8 ± 1.5	3.8 ± 1.5	0.101
Induction, n (%)					0.045
Basiliximab	217 (76.1)	22 (64.7)	174 (66.4)	50 (65.8)	
Anti-thymocyte globulin	68 (23.9)	12 (35.3)	88 (33.6)	26 (34.2)	
Main immunosuppressant, n (%)					
Tacrolimus: Cyclosporine	278: 6	34: 0	259: 3	76: 0	0.733
PRA > 50%	32 (19.5)	4 (20.0)	27 (13.9)	6 (15.4)	0.491

Values are expressed as means ± SDs, n (%).

KDPI, kidney donor profile index; KTR, kidney transplant recipient; CVA, cerebrovascular accident; ESRD, end-stage renal disease, HLA, human leukocyte antigen; PRA, panel reactive antibody.

### Comparison of short-term and long-term clinical outcomes according to the KDPI score in deceased donors and the age of kidney transplant recipients

**Figure 1:**
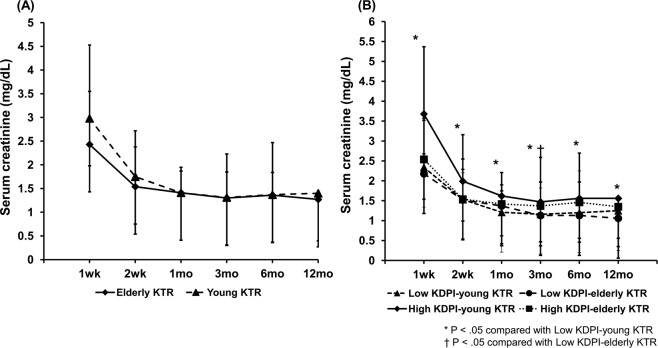
Comparison of the changes of allograft function (serum creatinine level) after KT **(A)** between the elderly KTR and young KTR groups and **(B)** among the four subgroups (low KDPI-young KTR, low KDPI-elderly KTR, high KDPI-young KTR and high KDPI-elderly KTR subgroup). *P < 0.05 vs. low KDPI-young KTR, †P < 0.05 vs. low KDPI-elderly KTR. Abbreviations: KT, kidney transplantation; KTRs, kidney transplant recipients; KDPI, kidney donor profile index.

In the short-term clinical outcomes, the incidence of delayed graft function (DGF), biopsy-proven acute rejection (BPAR) within 1 year after KT, BK-virus associated nephropathy (BKVN), cytomegalovirus infection (CMV infection), *Pneumocystis Jiroveci* pneumonia (PJP) were not significantly different among the four subgroups. In the long-term clinical outcomes, the incidence of cardiovascular diseases was the highest in the high KDPI-elderly KTR group compared to other groups (*P* = 0.011), but there were no significant differences in the rate of late acute rejection, chronic antibody-mediated rejection (cAMR), chronic allograft dysfunction (CAD), biopsy-proven calcineurin inhibitor toxicity, and malignancies among the 4 groups (Table 2). Allograft function assessed by serum creatinine level within 12 months post-KT did not differ between the elderly KTR group and the young KTR group (Fig. 1A). In the four subgroup analysis, allograft function within 12 months post-KT was the lowest in the high KDPI-young KTR subgroups compared to other subgroups (*P* < 0.05) (Fig. 1B).

**Table 2:** Comparison of short-term and long-term outcomes according to KDPI score and the age of KTRs.

Variables	Low KDPI-young KT	Low KDPI-elderly KT	High KDPI-young KT	High KDPI-elderly KT	p for Trend
Short-term outcomes, n (%)					
Delayed graft function	53 (18.6)	5 (14.7)	49 (18.7)	12 (15.8)	0.924
Biopsy-proven acute rejection	38 (13.3)	2 (5.9)	36 (13.7)	7 (9.2)	0.520
BK virus-associated nephropathy	3 (1.1)	0 (0)	8 (3.1)	3 (3.9)	0.203
CMV infection	46 (16.1)	6 (17.6)	36 (13.7)	18 (23.7)	0.224
PJP pneumonia	6 (2.1)	0 (0)	9 (3.4)	4 (5.3)	0.329
Long-term outcomes, n (%)					
Late acute rejection	18 (26.1)	2 (16.7)	13 (22.4)	3 (30.0)	0.850
Chronic antibody mediated rejection	3 (1.1)	1 (2.9)	1 (0.4)	1 (1.3)	0.244
Chronic allograft dysfunction	12 (4.2)	0	15 (5.7)	3 (3.9)	0.592
Biopsy-proven CNI-toxicity	20 (7.0)	0	11 (4.2)	1 (1.3)	0.099
Cardiovascular diseases	43 (15.1)	2 (5.9)	27 (10.3)	18 (23.7)	0.011
Malignancies	11 (4.0)	1 (3.1)	6 (2.5)	7 (9.2)	0.077

Values are expressed as means ± SDs, n (%).

KDPI, kidney donor profile index; KTR, kidney transplant recipient; CMV, cytomegalovirus; PJP, pneumocystis jiroveci pneumonia; CNI, calcineurin inhibitor.

### Comparison of the death-censored allograft survival according to the KDPI score in deceased donors and the age of kidney transplant recipients

**Figure 2:**
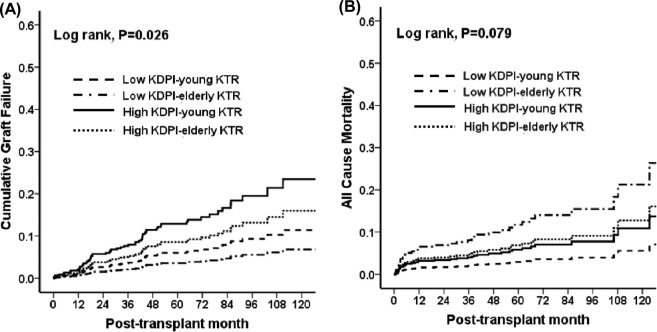
Comparison of **(A)** the death-censored allograft survival rate (vs. low KDPI-young KTR; low KDPI-elderly KTR, HR 0.600, 95% C.I. 0,078-4.379, P = 0.584 for; high KDPI-young KTR, HR 2.220, 95% C.I. 1.252-3.936, P = 0.006; high KDPI-elderly KTR, HR 1.441, 95% C.I. 0.533-3.893, P = 0.472) and **(B)** the patient survival rate (vs. low KDPI-young KTR; low KDPI-elderly KTR, HR 4.177, 95% C.I. 1.304-13.379, P = 0.016; high KDPI-young KTR, HR 2.013, 95% C.I. 0.918-4.416, P = 0.081; high KDPI-elderly KTR, HR 2.386, 95% C.I. 0.809-7.036, P = 0.115) among the low KDPI-young KTR, low KDPI-elderly KTR, high KDPI-young KTR and high KDPI-elderly KTR subgroups. Abbreviations: KDPI, kidney donor profile index; KTRs, kidney transplant recipients; HR, hazard ratio; C.I., confident interval.

A total of 60 cases (60/657, 9.1%) of allograft failure developed, including 18 patients in the low KDPI-young KTR (18/285, 6.3%), 36 patients in the high KDPI-young KTR (36/262, 13.7%) 1 patient in the low KDPI-elderly KTR (1/34, 2.9%), 5 patients in the high KDPI-elderly-KTR subgroup (5/76, 6.6%). No significant difference was detected in the distribution of the causes of allograft failure among the four subgroups (Table 3). In comparison of allograft survival rate using Cox regression analysis hazard model, high KDPI-young KTR subgroup showed the lowest allograft survival compared to other subgroups (*P* = 0.026) (Fig. 2A). When the risks for allograft failure were evaluated using the low KDPI-young KTR subgroup as the reference group, the high KDPI-young KTR subgroup had the highest risk of allograft failure after adjustment for DGF, transplant years (1996~2005 vs. 2006~2010 vs. 2011~2017), transplant centers, prior KT, DM of KTRs, HLA mismatch number, high PRA (>50%), the Chronic Kidney Disease Epidemiology Collaboration (CKD-EPI) eGFR at 12 months after KT, sex, age of DDs, and acute rejection. There was a significant interaction between high-KDPI scores in donors and young KTRs in the risk for the allograft failure (p for interaction = 0.004) (Table 4).

**Table 3:** Comparison of the causes of graft failure and death according to KDPI score and the age of KTRs.

Variables	Low KDPI-young KT	Low KDPI-elderly KT	High KDPI-young KT	High KDPI-elderly KT	p for Trend
Causes of graft failure, n (%)					0.204
Acute rejection	5 (28)	0	6 (17)	0	
Chronic allograft dysfunction	11 (61)	0	19 (53)	3 (60)	
Chronic antibody mediated rejection	0	0	2 (1)	0	
Recurrent glomerulonephritis	0 (0)	0	5 (14)	0	
BK virus-associated nephropathy	1 (6)	1 (100)	3 (8)	2 (40)	
Unknown	1 (6)	0	1 (3)	0	
Causes of death, n (%)					0.172
Cardiovascular disease	0	0	6 (37.5)	0	
Infection	6 (50.0)	2 (50)	3 (17.6)	4 (80)	
Malignancy	2 (16.7)	0	3 (17.6)	0	
Hepatic failure	2 (16.7)	0	1 (5.9)	0	
Unknown	2 (8.3)	2 (25)	4 (5.9)	1 (20)	

Values are expressed as means ± SDs, n (%).

KDPI, kidney donor profile index; KTR, kidney transplant recipients.

**Table 4:** Hazard ratios of death-censored allograft failure according to KDPI score and the age of KTRs.

	Unadjusted HR (95% C.I.)	P	Adjusted HR^a^ (95% C.I.)	P	P-value for interaction
Low KDPI-young KTR	Reference		Reference		0.004
Low KDPI-elderly KTR	0.584 (0.078–4.379)	0.600	1.316 (0.165–10.487)	0.795	
High KDPI-young KTR	2.220 (1.252–3.936)	0.006	2.653 (1.098–6.415)	0.030	
High KDPI-elderly KTR	1.441 (0.533–3.893)	0.472	1.792 (0.514–6.250)	0.360	

^a^Adjusted by DGF, transplant years (1996~2005 vs. 2006~2010 vs. 2011~2017), transplant centers, prior KT, DM of KTRs, HLA mismatch number, high PRA (>50%), eGFR calculated by CKD-EPI equation at 12 months after KT, sex, age of donor, acute rejection.

Abbreviations: KDPI, kidney donor profile index; KTR, kidney transplant recipient; HR, hazard ratio; DM, diabetes mellitus; PRA, panel reactive antibody; eGFR, estimated glomerular filtration rate; CKD-EPI, Chronic Kidney Disease Epidemiology Collaboration.

### Comparison of the patient survival according to the kdpi score in deceased donor and the age of kidney transplant recipients

A total of 38 patients (38/657 (5.8%)) died, of which 12 patients in the low KDPI-young KTRs (12/285, 4.2%), 17 patients in the high KDPI-young KTRs (17/262, 6.5%), 4 patients in the low KDPI-elderly KTRs (4/34, 11.8%) and 5 patients in the high KDPI-elderly KTRs (5/76, 6.6%) subgroups. There was no significant difference in the distribution of the causes of patient death among the four subgroups (Table 3). Patient survival rate was the lowest in the low KDPI-elderly KTR subgroup compared to other subgroups (especially, comparing low KDPI-young KTR, HR 4.177, 95% C.I. 1.304-13.379, *P* = 0.016) (Fig. 2B). When the risks for patient death were evaluated using the low KDPI-young KTR subgroup as the reference group, there was no significant difference among the four subgroups after adjustment for DGF, transplant years (1996~2005 vs. 2006~2010 vs. 2011~2017), prior KT, DM of KTRs, HLA mismatch number, high PRA (>50%), the CKD-EPI eGFR at 12 months after KT, sex, age of DDs, acute rejection and malignancy. There was no significant interaction between high-KDPI scores in donors and young KTRs in the risk for the patient death (p for interaction = 0.420) (Table 5).

**Table 5:** Hazard ratios of all-cause mortality according to KDPI score and the age of KTRs.

	Unadjusted HR (95% C.I.)	P	Adjusted HR^a^ (95% C.I.)	P	P-value for interaction
Low KDPI-young KTR	Reference		Reference		0.420
Low KDPI-elderly KTR	4.177 (1.304–13.379)	0.016	2.841 (0.314–25.737)	0.353	
High KDPI-young KTR	2.013 (0.918–4.416)	0.081	1.590 (0.509–4.962)	0.425	
High KDPI-elderly KTR	2.386 (0.809–7.036)	0.115	2.870 (0.636–12.961)	0.170	

^a^Adjusted by DGF, transplant years (1996~2005 vs. 2006~2010 vs. 2011~2017), prior KT, DM of KTRs, HLA mismatch number, high PRA (>50%), eGFR calculated by CKD-EPI equation at 12 months after KT, sex, age of donor, acute rejection, malignancy.

Abbreviations: KDPI, kidney donor profile index; KTR, kidney transplant recipient; HR, hazard ratio; DGF, delayed graft function; KT, kidney transplantation; DM, diabetes mellitus; HLA, human leukocyte antigen; PRA, panel reactive antibody; eGFR, estimated glomerular filtration rate; CKD-EPI, Chronic Kidney Disease Epidemiology Collaboration.

## Discussion

In our previous study, we found that high KDPI scores in DDs can be associated with adverse allograft outcomes^12^. However, in that study, we did not consider recipient factors, which can also interact with donor factors to affect allograft outcomes^9^. In this study, we investigated if KT from DDs with high KDPI scores had differential impacts on the recipients according to their age. We found that the impact was less significant in elderly KTRs than in young KTRs. This suggests that DDs with high KDPI scores can be used for appropriate recipients such as elderly recipients rather than being discarded.

First, we compared the impact of high KDPI scores in DDs on short-term clinical outcomes according to the age of KTRs. In contrast to our expectation, there was no significant difference in the incidence of DGF among the four subgroups, which suggests that high KDPI scores in DDs did not increase the risk for DGF. In previous reports, including ours, acute kidney injury (AKI) defined by any criteria was a significant risk factor for DGF^11,13,15,16^. Even though the KDPI scoring system included serum creatinine as one of the variables, serum creatinine has limited ability to represent the dynamic pattern of AKI^17–19^. In addition, other variables constituting the KDPI score may be associated with chronic changes, rather than AKI, in donor kidneys. In contrast, allograft functions within a year after KT were the lowest in the high KDPI-young KTR subgroup. Baseline capacity to recover may be lower in kidneys with high KDPI score than kidneys with low KDPI score because of the underlying chronic change as we mentioned above^20^. However, the metabolic demands may be higher in young-KTRs than elderly-KTRs. Therefore, this physiological mismatch between donors and recipients may result in the lowest allograft function in the high KDPI-young KTR subgroup.

Our main interest was if DDs with high-KDPI scores had different impacts on long-term allograft survival in the elderly KTR and young KTR groups. Of note, the high KDPI-young KTR subgroup showed worse allograft outcomes compared to the other groups. Additionally, young-age recipients showed significant interaction with high KDPI scores in DDs. Hence, in multivariate analysis using the Cox regression hazard model, the co-existence of high-KDPI DDs and young KT recipients was an independent contributor to allograft failure. These findings suggest that high KDPI in DDs may be a significant risk factor for allograft failure in young KTRs but not in elderly KTRs. Along with the lowest allograft function in the high KDPI-young KTR subgroup, the reason for the findings can be explained by the significance of physiological matching of the nephron mass between donors and recipients. Indeed, mismatch between metabolic demand and nephron mass can result in adverse allograft outcomes^21^ and can explain why allograft outcome can be worse when kidneys from smaller donors were donated to relatively larger recipients—because a small body may have a smaller nephron mass^22–25^. In the case of KT with elderly kidney donors especially, age-matching is also a main factor in post-transplant clinical outcomes because increases in the functional loss of nephrons in an elderly donor may not meet the metabolic demand of younger recipients^26,27^. Consistent with this, high KDPI score in DDs who are older and may have more chronic kidney injury can have a smaller kidney nephron mass. All these findings suggest that when young recipients who may have a higher metabolic demand, receive kidneys from DDs with high KDPI scores who may have smaller nephron mass, the long-term allograft outcome can be worse than that in patients who receive kidneys from DDs with low KDPI scores.

In contrast to our expectation, the proportion of chronic allograft dysfunction as the cause of allograft failure was not different among the four subgroups. At first, we expected that allograft failure due to physiologic mismatch may develop as non-specific “chronic allograft nephropathy” instead of an immunologic process, such as acute rejection or chronic antibody-mediated rejection. However, even though allograft rejection could be a direct cause of allograft failure in some patients, when it develops in kidneys with lower baseline capacity, such as kidneys from high-KDPI donors, the probability for allograft failure can increase. Indeed, in transplant naive patients, AKI that develops in chronic kidney disease can show adverse outcome in terms of the development of ESRD^28,29^. Taken together, we thought that the main reason for the adverse allograft outcome in the high-KDPI-DDKT subgroup may have resulted from the low kidney capacity at baseline, which induced a physiologic mismatch between the donor and recipients in this group. In contrast to death-censored allograft survival, the patient survival rate did not show significant difference after adjustment by confounding factors. In addition, high KDPI scores in DDs did not show significant interaction with the age of KTRs. (Table 5) The reason for this is unclear, but it may be because recipient factors, rather than donor factors, are more important for patient mortality, as shown in previous studies. Therefore, the quality of the donor kidney was not found to be a significant factor^30–33^.

Our study had limitations that we suggested in our previous reports using this cohort. First, not all KTRs corresponding to donors included in this study were included in this analysis because some organs were transferred to another institution according to the organ distribution rule in Korea, which could have induced a bias during analysis. To reduce this kind of limitation, we added more transplant centers to this study than in the previous report. Second, we could not differentiate cAMR from non-specific chronic allograft dysfunction because allograft biopsies were performed in only four of 44 patients who showed indolent courses due to allograft failure, of which two biopsy findings were compatible with cAMR. Serial monitoring of HLA-DSA and extensive surveillance allograft biopsies may help to clarify this issue. Lastly, the sample size of elderly group is low, hence there is possibility that the findings could be altered with a larger cohort.

In conclusion, the impact of high KDPI scores in DDs on allograft survival may be less significant in the elderly KTR group maybe because of physiologic matching of the nephron mass between donors and recipients in this group. Our finding suggests that even DDs with high KDPI scores should not be discarded and can be considered for transplantation for KTRs who need relatively smaller nephron mass, such as elderly patients.

## Materials and Methods

### Study population

Between October 1996 and December 2017, 714 cases of deceased donor kidney transplantation (DDKT) were performed at four transplant centers (Seoul St. Mary’s hospital, Keimyung University Dongsan Medical Center, Uijeongbu St. Mary’s Hospital, and Daejeon St. Mary’s Hospital). Among them, we included 657 KTRs who received kidneys from 526 DDs, in whom the data for the calculation of KDPI scores were available. We collected data about the DDs that included age, height, weight, ethnicity, history of HTN or DM, cause of death, serum creatinine, hepatitis C virus serology, and donation after cardiac death. The KDPI scores were calculated using the KDPI calculator on the Organ Procurement and Transplantation Network website using these 10 items^34–36^. We defined high KDPI scores as 65% of the KDPI, the median KDPI score in this cohort. Thus, among 526 DDs, 257 (48.9%) were classified as high-KDPI donors and 269 (51.1%) were classified as low-KDPI donors in this study.

**Figure 3:**
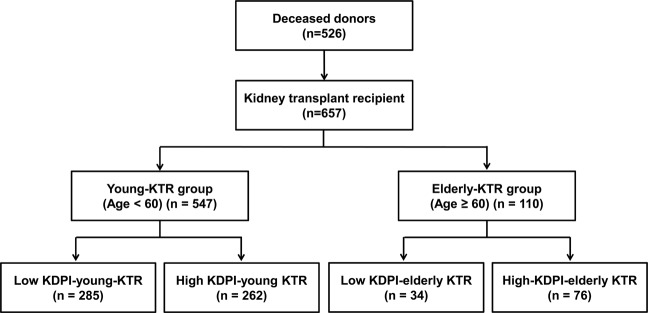
Patient algorithm and distribution in this study. KTRs were classified into elderly KTR and young KTR groups based on an age of 60. Each group was subdivided into high-KDPI and low-KDPI subgroups based on a median KDPI value of 65% in the corresponding DDs. Abbreviations: KTRs, kidney transplant recipients; KDPI, kidney donor profile index; DDs, deceased donors.

The patient distributions in the various groups are presented in Fig. 3. Elderly KTR was defined as an equal or older than 60 years according to previous report^37^. Therefore, a total of 657 KTRs from 526 DDs were divided into elderly KTR and young KTR groups based on an age of 60. Thus, 547 (83.3%) were classified into the young KTR group and 110 (16.7%) KTRs were classified into the elderly KTR group. Within the young KTR group, 285 cases (52.1%) were classified into the low KDPI-young KTR subgroup and another 262 cases (47.9%) were classified into the high KDPI-young KTR subgroup. Within the elderly KTR group, 34 KTRs (30.9%) received kidneys from DDs with low KDPI scores (low KDPI-elderly-KTR subgroup) and another 76 KTRs (69.1%) received kidneys from DDs with high KDPI scores (high KDPI-elderly KTR subgroup). The median follow-up period of this study was 60.3 (interquartile range 41.6 ~ 86.1) months and it did not differ among 4 subgroups.

### Clinical parameters and outcomes

We retrospectively analyzed the medical records of the donors and recipients. Age, sex, BMI, history of DM and HTN, cause of death, and serum creatinine, eGFR as an assessment of kidney function from the day of admission to the day of KT were included in the baseline donor data. Additionally, we collected the following baseline recipient data: age, sex, BMI, history and duration of dialysis before KT, number of previous KTs, cause of ESRD, history of DM and HTN, cold ischemic time, number of HLA mismatches, type of immunosuppressant used for induction and maintenance, and the percentage of PRAs.

We analyzed the short-term clinical outcomes, including the incidence of DGF, BPAR within 1 year after KT, BKVN, CMV infection, PJP, and the long-term clinical outcomes including the rate of late acute rejection, cAMR, CAD, biopsy-proven calcineurin inhibitor toxicity, cardiovascular diseases, and malignancies for all 4 groups of patients (among the low KDPI-young KTR, low KDPI-elderly KTR, high KDPI-young KTR and high KDPI-elderly KTR groups). Interpretation of the indicated or protocol biopsy findings was conducted according to the Banff classification 2009^38^. DGF was defined as the requirement for PD or HD within a week after KT^39^. BPAR was defined as acute T cell-mediated and antibody-mediated rejection by allograft biopsies according to the Banff classification. BKVN was diagnosed by allograft biopsies due to allograft dysfunction and BK viremia. CMV infection was defined as virus detection of viral proteins or nucleic acid in whole blood, including CMV disease diagnosed as the CMV infection with companying symptoms. PJP was confirmed by histological identification of the causative organism in sputum or bronchoalveolar lavage or by molecular analysis of PCR. Late acute rejection was defined as an acute rejection scored according to the last Banff classification from 1 year after KT. cAMR, and biopsy-proven calcineurin inhibitor toxicity were diagnosed by allograft biopsies. CAD was defined when i) the allograft biopsy findings showed non-specific chronic tissue injury without evidence of rejection or ii) an allograft biopsy was not done within one year of allograft failure and the allograft function showed a gradual deterioration several years before the allograft failure. Cardiovascular diseases were defined as coronary artery disease or cerebral infarction requiring hospitalization or intervention. Malignancies included both recurrence of malignancy before KT and de novo malignancy after KT.

We also investigated the changes in allograft function with serum creatinine within one year after KT, death-censored allograft survival, and patient survival rates among the 4 groups. Death-censored allograft survival was defined as the time between KT and the restart of dialysis or pre-emptive KT, except for death with a functioning allograft. Patient survival was defined as the time between KT and death from any cause.

The primary outcome of this study was a comparison of the impact of high KDPI scores in DDs on the death-censored allograft survival between elderly KTR and young KTRs. For this, we compared death-censored allograft survival among the low KDPI-young KTR, low KDPI-elderly KTR, high KDPI-young KTR and high KDPI-elderly KTR subgroups, and analyzed the interaction between recipient age and KDPI score in donors. Secondary outcomes included the incidence of DGF, BPAR within 1 year after KT, BKVN, CMV infection, PJP, late acute rejection, cAMR, CAD, calcineurin inhibitor toxicity, cardiovascular diseases, and malignancies; changes in allograft function determined by serum creatinine level and eGFR calculated using the CKD-EPI equation^40^ at one week, two weeks, one month, three months, six months, and 12 months after KT; the cause of allograft failure; the cause of patient death; and patient survival rate. The cause of allograft failure included biopsy-proven acute rejection (both T-cell mediated rejection and antibody-mediated rejection), biopsy-proven cAMR, CAN, biopsy-proven BKVN, and biopsy-proven recurrent primary glomerulonephritis.

This study was approved by the Institutional Review Boards of Seoul St. Mary’s Hospital (XC15RIMI0061K), Uijeongbu St. Mary’s Hospital (XC15RIMI0061U), Keimyung University School of Medicine, Dongsan Medical Center (2019-09-051), and Daejeon St. Mary’s Hospital (XC15RIMI0061K). The requirements for informed consent were waived by the Institutional Review Boards of four centers because the study was explained to all patients prior to transplantation, personal data related to the patient’s clinical course after transplant was used, and information identifying the individual was protected. This study was a retrospective medical record study and this manuscript did not contain personally identifiable information. In addition to this, all methods were performed in accordance with the relevant guidelines and regulations. The four transplant centers (Seoul St. Mary’s Hospital, Uijeongbu St. Mary’s Hospital, Keimyung University School of Medicine, Dongsan Medical Center, and Daejeon St. Mary’s Hospital) never underwent transplantation with kidneys procured from prisoners, and this study did not include them.

### Statistical analyses

Continuous variables with normal distribution were expressed as mean ± standard deviation and analyzed by Student’s t-tests. Continuous variables with non-normal distribution were expressed as median and interquartile range and were analyzed by the one way analysis of variance (one way ANOVA) test. Categorical variables were expressed as count and percentage and analyzed by the Chi-squared or Fisher’s exact test. Death-censored graft survival and patient survival rates were described and compared by Cox regression analysis. Cox proportional hazards regression analysis was used to investigate the impact of KDPI scores or KTR age on clinical outcomes of DDKT considering confounding variables, such as DGF, transplant years (1996~2005 vs. 2006~2010 vs. 2011~2017), transplant centers, prior KT, DM of KTRs, HLA mismatch number, high PRA ( > 50%), the Chronic Kidney Disease Epidemiology Collaboration (CKD-EPI) eGFR at 12 months after KT, sex, age of DDs, and acute rejection. All missing data were censored from the last follow-up date. Interaction effects between the KTR age (elderly vs. young) and KDPI scores (high vs. low) in the DDs were explored by adding the interaction terms to the model. P values <0.05 were statistically significant. All statistical analyses were performed using SPSS 21.0 software (SPSS Inc., Chicago, IL, USA) and the MedCalc statistical package version 15.5 (MedCalc, Mariakerke, Belgium).

## Supplementary information

Supplementary table 1.

## Data Availability

All data generated or analyzed during this study are included in this published article and its Supplementary Information files. The datasets generated during and/or analyzed during the current study are available from the corresponding author on reasonable request.
