# Supplementary material for: Impact of Kidney Donor Profile Index Scores on Post-Transplant Clinical Outcomes Between Elderly and Young Recipients, A Multicenter Cohort Study
Source: Sci Rep. 2020 Apr 24;10:7009. doi: 10.1038/s41598-020-64055-8 (PMC7181596; doi:10.1038/s41598-020-64055-8)
Supplement: Supplementary file 1 — Supplementary table 1. [file 41598_2020_64055_MOESM1_ESM.doc]

**Impact of Kidney Donor Profile Index Scores on Post-Transplant Clinical Outcomes Between Elderly and Young Recipients, A Multicenter Cohort Study**

Woo Yeong Park^8,9^, Jeong Ho Kim^3^, Eun Jung Ko^1,2^, Ji-Won Min ^1,4^, Tae Hyun Ban ^1,5^,

Hye-Eun Yoon ^1,6^, Young Soo Kim ^1,7^, Kyubok Jin ^8,9^, Chul Woo Yang ^1,2^, Seungyeup Han ^8,9*^ & Byung Ha Chung ^1,2*^

^1^Transplant research center, ^2^Division of Nephrology, Department of Internal Medicine, Seoul St. Mary's Hospital, College of Medicine, The Catholic University of Korea, Seoul, Republic of Korea. ^3^Division of Nephrology, Department of Internal Medicine, Daejeon St. Mary’s hospital, College of Medicine, The Catholic University of Korea, Daejeon, Republic of Korea.

^4^Division of Nephrology, Department of Internal Medicine, Bucheon St. Mary’s hospital, College of Medicine, The Catholic University of Korea, Bucheon, Republic of Korea. ^5^Division of Nephrology, Department of Internal Medicine, Eunpyeong St. Mary’s hospital, College of Medicine, The Catholic University of Korea, Seoul, Republic of Korea. ^6^Division of Nephrology, Department of Internal Medicine, Incheon St. Mary’s hospital, College of Medicine, The Catholic University of Korea, Incheon, Republic of Korea. ^7^Division of Nephrology, Department of Internal Medicine, Uijeongbu St. Mary’s hospital, College of Medicine, The Catholic University of Korea, Uijeongbu, Republic of Korea. ^8^Department of Internal Medicine, Keimyung University School of Medicine, ^9^Keimyung University Kidney Institute, Daegu, Republic of Korea. Correspondence and requests for materials should be addressed to B.H.C. (email: chungbh@catholic.ac.kr) or S.H. (email: shyi@kigam.re.kr)

***These Corresponding authors equally contributed to this article**

***Correspondence to Byung Ha Chung, MD, PhD**

Department of Internal Medicine, Seoul St. Mary’s Hospital, 505 Banpo-Dong, Seocho-Ku, 137-040, Seoul, Korea. Fax: +82-2-536-0323, Phone: +82-2-2258-6066,

E-mail ; chungbh@catholic.ac.kr

***Correspondence to Seungyeup Han, MD, PhD**

Department of Internal Medicine, Keimyung University School of Medicine,

Keimyung University Kidney Institute,

1035 Dalgubeol-daero. Dalseo-gu, Daegu 42601, Korea. Tel: +82-53-258-7712

Fax: +82-53-258-4739, E-mail: hansy@dsmc.or.kr

**Table S1. Comparison of clinical and laboratory parameters between elderly-KTR group and young-KTR group**

| **Variable** | **Elderly-KT**  **(n=91)** | **Young-KT**  **(n=435)** | ***P-*value** |
| --- | --- | --- | --- |
| **Donors** |  |  |  |
| Age at KT (years) | 50.9 ± 15.0 | 43.8 ± 14.1 | <0.001 |
| Gender (Male:Female) | 63:28 | 305:130 | 0.900 |
| Body mass index (kg/m^2^) | 23.0 ± 3.0 | 23.1 ± 3.7 | 0.842 |
| Hypertension, n (%) | 22 (24.2) | 85 (19.5) | 0.319 |
| Diabetes mellitus, n (%) | 10 (11.0) | 40 (9.2) | 0.560 |
| Cause of donor death – CVA, n (%) | 67 (73.6) | 296 (68.0) | 0.321 |
| Baseline eGFR(ml/min/1.73m^2^)(CKD-EPI) | 116.2 ± 36.7 | 117.9 ± 33.8 | 0.672 |
| Acute kidney injury, n (%) | 57 (62.6) | 236 (54.3) | 0.164 |
| High KDPI, n (%) | 61 (67.0) | 196 (45.1) | <0.001 |
|  | **Elderly-KTR**  **(n=110)** | **Young-KTR**  **(n=547)** | ***P-*value** |
| **Recipient** |  |  |  |
| Transplant year, n (%) |  |  | 0.024 |
| 1996 ~ 2005 | 0 | 8 (1.5) |  |
| 2006 ~ 2010 | 7 (6.4) | 88 (16.1) |  |
| 2011 ~ 2017 | 53 (93.6) | 316 (82.4) |  |
| Age at KT(year) | 63.6 ± 3.0 | 46.7 ± 8.5 | <0.001 |
| Gender (Male:Female) | 66:44 | 323:224 | 0.915 |
| Body mass index (kg/m^2^) | 23.4 ± 3.1 | 23.1 ± 3.9 | 0.528 |
| Hypertension, n (%) | 97 (88.2) | 456 (83.4) | 0.252 |
| Diabetes mellitus, n (%) | 27 (24.5) | 110 (20.1) | 0.305 |
| Dialysis duration, years | 6.9 ± 4.1 | 8.6 ± 11.1 | 0.114 |
| Previous KT, n (%) | 7 (6.4) | 63 (11.5) | 0.128 |
| Cause of ESRD, n (%) |  |  | 0.127 |
| Glomerulonephritis | 45 (40.9) | 253 (46.3) |  |
| Diabetes mellitus | 28 (25.5) | 86 (15.7) |  |
| Hypertension | 18 (16.4) | 97 (17.7) |  |
| Others | 19 (17.3) | 111 (20.3) |  |
| Cold ischemic time (min) | 251.8 ± 127.9 | 250.8 ± 123.5 | 0.941 |
| HLA mismatch number | 3.7 ± 1.6 | 3.6 ± 1.5 | 0.568 |
| Induction, n (%) |  |  | 0.209 |
| Basiliximab | 72 (65.5) | 391 (71.5) |  |
| Anti-thymocyte globulin | 38 (34.5) | 156 (28.5) |  |
| Major immunosuppressant  Tacrolimus : Cyclosporine | 110 : 0 | 537 : 9 | 0.475 |
| PRA > 50 %, n (%) | 10 (16.9) | 59 (16.5) | 1.000 |

Values are expressed as means ± SDs, n (%). eGFR is calculated using MDRD formula.

KDPI, kidney donor profile index; KT, kidney transplantation; eGFR, estimated glomerular filtration rate; CVA; cerebrovascular accident; CKD, chronic kidney disease; ESRD, end-stage renal disease; HLA, human leukocyte antigen; PRA, panel reactive antibody
